# Effectiveness of a mindfulness program in changing attitudes to reduce work stress: a randomized controlled trial

**DOI:** 10.3389/fpubh.2026.1912184

**Published:** 2026-09-22

**Authors:** Raúl Guzmán, Yuri Félix Chávez-Luque, Guillermo Sebastián Medina, Celin Daniel Valdiviezo

**Affiliations:** Professional School of Psychology, Universidad Católica de Santa Maria, Arequipa, Peru

**Keywords:** industrial workers, mindfulness, occupational accidents, occupational safety, work stress

## Abstract

Worker safety is fundamental in any organization, so we propose a mindfulness-based program enhanced by virtual reality (VR) to improve attitudes toward preventing occupational risks and accidents and reduce work-related stress in an industrial company. The program was implemented as a complement to Behavior-Based Safety (BBS) of nine 30-min sessions delivered in person and continuously by two researchers; VR was used in three of the nine sessions. Each session was divided into motivation, knowledge development, transfer, evaluation, and closure sections. The control group worked only with BBS, a standard methodology designed to reduce workplace accidents and injuries through systematic observation and intervention in worker behavior. A randomized block design (RBD) was used with a sample of 34 workers from high-risk industrial areas, who were matched by their sociodemographic characteristics and randomly assigned to one of the two groups. An attitude scale regarding occupational risk prevention (accidents) and a work-related stress scale were used for the assessment. The results showed a significant improvement in attitudes toward occupational risk prevention (*p* < 0.005) and a reduction in work-related stress, although this finding was not statistically significant according to MANOVA and Bonferroni correction. In conclusion, this study shows that mindfulness complements and significantly improves attitudes toward occupational risk prevention.

## Highlights

**Public health relevance—How does this work relate to a public health issue?**

- This study addresses occupational risk prevention, a key public health priority centered on reducing workplace injuries, accidents, and morbidity among workers.
- By examining an intervention designed to decrease work-related stress, it tackles a well-established determinant of physical and mental health, contributing to broader efforts to improve worker wellbeing.

**Public health significance—Why is this work of significance to public health?**

- The findings show that a mindfulness-based program enhanced by virtual reality can improve attitudes toward occupational risk prevention, providing evidence for innovative strategies consistent with public health approaches to injury prevention.
- Through a randomized controlled trial, this study offers rigorous empirical data that can benefit evidence-based interventions and policies aimed at strengthening occupational safety and psychosocial health.

**Public health implications—What are the key implications or messages for practitioners, policy makers, and/or researchers in the field of public health?**

- For occupational health practitioners, the results indicate that brief mindfulness programs can be integrated into frameworks such as Behavior-Based Safety to strengthen preventive safety cultures in high-risk industries.
- For policymakers and researchers, this study highlights the value of investing in novel psychosocial interventions—such as mindfulness combined with virtual reality—to improve workplace safety and reduce stress-related burdens, even when some outcomes do not reach statistical significance.

## 1 Introduction

This research is framed within a key area of human resource management: occupational health and safety. In this context, the traditional approach to preventing workplace accidents and occupational diseases must be broadened to include comprehensive wellbeing and quality of work life (1).

Organizations can enhance their competitiveness by promoting the strategic value of people through the recognition of human capital (2). The most competitive organizations tend to focus on developing advanced constructs, such as psychological capital in the workplace (3). This perspective can be further strengthened through the application of positive psychology in occupational health and safety, contributing to the development of healthy organizations, employee wellbeing, organizational effectiveness, and resilience (4, 5).

The psychological approach to occupational health and safety, based on these perspectives, has shown significant results, particularly through the development of behavioral programs such as Behavior-Based Safety (BBS), conceived as an effective strategy to improve safety culture while reducing workplace accidents. This approach emphasizes the active and engaged participation of employees in promoting safe behaviors (6). Subsequently, this model was expanded through the Values-Based Safety approach, which establishes that organizational climate is closely related to safe behavior (7). In addition, researchers have examined how safety attitudes (8) and safety climate influence safe behaviors and, consequently, the prevention of workplace accidents (9).

Behavior-Based Safety (BBS) programs are widely used in high-risk industries. However, they face several challenges, such as the limited sustainability of behavioral change, observation fatigue, resistance to change, emotional strain under stress, and divided attention. In contrast, mindfulness promotes present-moment awareness, sensitivity to internal and external stimuli, emotional regulation, and stress reduction (10). Virtual reality (VR) provides an immersive environment that enhances attention, presence, realism, and engagement, enabling more realistic, safe, repeatable, and experiential training. The combination of mindfulness and VR has begun to be evaluated—with promising results—in relation to mental health, stress management, and safe work behaviors (10). In a mining study conducted in Peru, a group of high-risk workers undergoing a traditional BBS program was compared with another group participating in a VR-supported mindfulness program. A significant improvement in safe behavior indicators was observed in the experimental group (Mindfulness + VR) compared to the BBS group (10, 11).

It is important to note that mindfulness is not intended to replace BBS, but rather to enhance it by incorporating an internal psychological dimension. Together, these strategies form a more comprehensive approach to safety: external–behavioral (BBS) and internal–cognitive–emotional (mindfulness).

More recently, mindfulness has been incorporated into the field of occupational safety, standing out for its benefits in improving workplace health and safety, particularly in reducing stress, increasing concentration, and promoting employee wellbeing (12). Likewise, the use of virtual reality and its potential applications in this field have been explored (13).

The concept of mindfulness, developed and popularized by Kabat-Zinn (14), represents a modern approach in the field of health and wellbeing. It has gained increasing support in disciplines such as neuroscience (15) and has become especially influential in psychotherapy through its application in mindfulness-based stress reduction (14). Mindfulness practice has established itself as a key enabler across various domains, including health, education, and sports, and has expanded into organizational settings to improve safety and reduce occupational risks. It enables individuals to develop comprehensive awareness of internal and external mental processes, thereby contributing to the regulation of workers' behavior (16). Furthermore, mindfulness practices are associated with reduced stress—particularly during organizational transitions—while enhancing wellbeing, objectivity, and cognitive flexibility, which are crucial in the modern world (17). Empirical evidence supports these claims; for instance, (18) found that employees with higher levels of mindfulness committed fewer safety violations, demonstrating an inverse relationship between mindfulness and deviations from safety protocols. Consequently, mindfulness programs have been shown to reduce risky behaviors among employees, regardless of factors such as age, intelligence, or work experience (19).

Moreover, mindfulness has proven highly effective in preventing physical and mental disorders such as stress, depression, anxiety, and eating disorders (20). In organizational contexts, mindfulness is associated with reduced work-related stress, fostering a focus on the present moment and preventing over uncontrollable outcomes. This practice enables individuals to distinguish environmental stressors from their emotional responses, promoting more effective coping strategies (17). Present-moment awareness facilitates adaptation to stress, regardless of emotional states or perceived threat intensity (21). Mindfulness-based interventions, such as mindfulness-based stress reduction programs, have demonstrated effectiveness in high-stress occupations (22), and mindfulness has been identified as a predictor of safe work behaviors (23). Additionally, it is associated with lower emotional exhaustion and higher job satisfaction (12, 24), highlighting its value in enhancing organizational wellbeing and employee engagement. Mindful employees, by recognizing distractions, can refocus their attention on the task at hand, contributing to improvements in both psychological wellbeing and physiological indicators, such as respiratory and heart rates (25).

Mindfulness is defined as non-judgmental, moment-to-moment awareness, characterized by open, non-reactive, and unbiased attention (26). The mindfulness program considered in this study integrates five core components related to occupational safety. First, attention to the present moment facilitates early detection of warning signs, environmental anomalies, and unsafe conditions, thereby improving situational awareness. Second, openness to experience supports the clear communication of internal states and risk perceptions, which is critical for team safety. Third, acceptance is directly linked to the reduction of human error, as it enhances attention to critical tasks, reduces distraction-related accidents, and improves compliance with safety protocols. Fourth, letting go (detachment) fosters openness to safety feedback without defensiveness and contributes to a climate of psychological safety, where workers can report errors without fear. Finally, intentionality strengthens emotional regulation in stressful situations, potentially preventing impulsive responses that could compromise safety.

To support these components, key techniques are employed, including mindful breathing, which serves as the foundation of meditation and enhances awareness of thoughts; guided imagery, which uses the five senses to generate mental representations; and body scan practices (guided body awareness), which focus attention on physical sensations to promote relaxation and bodily awareness. These practices are essential for cultivating mindfulness and applying it to everyday life improvement (27).

There are, however, inconsistent findings regarding the relationship between mindfulness and safety, including studies that have found no supporting evidence. For example, a hospital-based study examining the implementation of a mindfulness program among nursing staff found no reduction in workplace accidents among healthcare professionals (28).

Nevertheless, attitudes toward occupational risk and accident prevention encompass individuals' beliefs, emotions, and predispositions regarding safety measures and policies, significantly influencing behavior in safety-critical environments. According to Qian and He (29), safety attitudes include subjective evaluations—such as the prioritization of safety—and can critically mediate the relationship between knowledge and behavior. Similarly, Ji (30) emphasized that in industrial settings, workers' attitudes toward risk prevention not only reflect their beliefs about safety procedures but also shape their behaviors, which are essential for ensuring workplace safety. These findings indicate that positive safety attitudes can reduce unsafe behaviors, thereby decreasing accidents and improving overall compliance with safety regulations.

Despite the growing evidence supporting mindfulness-based interventions for reducing workplace stress and improving employee well-being, important gaps remain regarding their application to occupational safety in high-risk industrial settings. In particular, limited evidence is available on whether a mindfulness-based intervention integrated with Behavior-Based Safety (BBS) can simultaneously influence workers' attitudes toward occupational risk and accident prevention and their work-related stress.

Although mindfulness has been associated with safer work behaviors and improved attentional and emotional regulation, most previous research has examined these outcomes separately rather than as complementary dimensions of occupational safety. Similarly, virtual reality (VR) has increasingly been incorporated into occupational safety training because of its ability to provide immersive, realistic, and safe simulations of hazardous situations; however, its integration as an experiential component of mindfulness-based safety training remains insufficiently explored. Recent evidence indicates that VR can enhance safety training outcomes, including safety motivation, risk perception, learning, and safety-related performance, but further research is needed to determine how it can be integrated with psychological and behavioral safety approaches in high-risk work environments (46, 47).

Therefore, the present study addresses this gap by examining a mindfulness-based program enhanced with VR as a complement to BBS, with the specific purpose of determining whether this combined approach can improve attitudes toward occupational risk and accident prevention while reducing work-related stress among workers in high-risk industrial areas. The study thus contributes to the literature by integrating behavioral, cognitive-emotional, and experiential dimensions of occupational safety within a single intervention framework.

The objective of this study was to determine the effectiveness of a mindfulness-based program enhanced by virtual reality in improving attitudes toward occupational risk and accident prevention, as well as reducing work-related stress, in a group that also received Behavior-Based Safety (BBS) training. This group was compared to a control group that received only BBS training. Additionally, the study aimed to assess whether mindfulness can enhance BBS, the standard method used by industrial organizations to improve safety and reduce workplace accidents.

## 2 Materials and methods

### 2.1 Method

#### 2.1.1 Participants

We employed a randomized controlled trial, specifically a randomized block design (40). The final sample size was calculated using gpower 3.1.9.4 software, with 80% statistical power and a high effect size for before-and-after mean comparisons. The suggested sample size was 12 workers for the experimental group. We ultimately decided to consider 20 workers in case of dropout, which did occur. All were employees of a transnational industrial company with homogeneous characteristics according to their age, sex, educational level, marital status, and work area, and all agreed to participate voluntarily. Initially, we gained access to information on all 250 employees of the company; managerial and administrative positions were then eliminated in order to focus on areas featuring maximal exposure to occupational risk due to accidents, such as maintenance, production, processes, and the supply chain. Informed consent was then presented, with the purpose of the research explained. Of those who agreed to participate voluntarily, a total of 40 workers were selected and randomly assigned to one of two groups. These groups were homogeneous: there was a control group that was subjected to the standard Behavior-Based Safety (BBS) method and an experimental group that was subjected to our mindfulness-based program, enhanced by virtual reality, in addition to the BBS standard. The company provided the facilities and conveniences for the research, and since the schedules rotated between morning and night, the intervention had to be adapted to a schedule that allowed everyone to attend. The randomly chosen sample consisted of 40 workers between 21 and 69 years of age, with an average age of 39.79 years (SD = 11.99). The sample was 80% men and 20% women. In total, 60% completed secondary school, and 40% completed technical school. Overall, 35% were single, and 65% were married/cohabiting. Regarding areas of work, 10%, 15%, 40%, and 35% came from the maintenance area, processes, production, and the supply chain, respectively. The final sample consisted of 34 workers with the following characteristics: The control group (BBS) was composed of 20 workers with an average age of 39.7 (SD = 10.64), of which 80% (16) were men and 20% (4) were women, while 60% (12) had completed secondary education and 40% (8) had technical training. In total, 35% (7) were single, and 65% (13) were married or in a free union, while 10% (2) were from the maintenance area, 15% (3) were from the process area, 40% (8) were from the production area, and 35% (7) were from the supply chain area. The experimental group (mindfulness) consisted of 20 workers, but in the end, only 14 remained. The mean age was 43.0 (SD = 12.89). In total, 86% (12) were men, and 14% (2) were women; 50% (7) had completed secondary education, and 50% (7) had technical training; 28.6% (4) were single, while 71.4% (10) were married or in a common-law relationship; and 14% (2) were from the maintenance area, 14% (2) came from the process area, 50% (7) were from the production area, and 22% (3) were from the supply chain area (Figure 1; Table 1).

**Figure 1:**
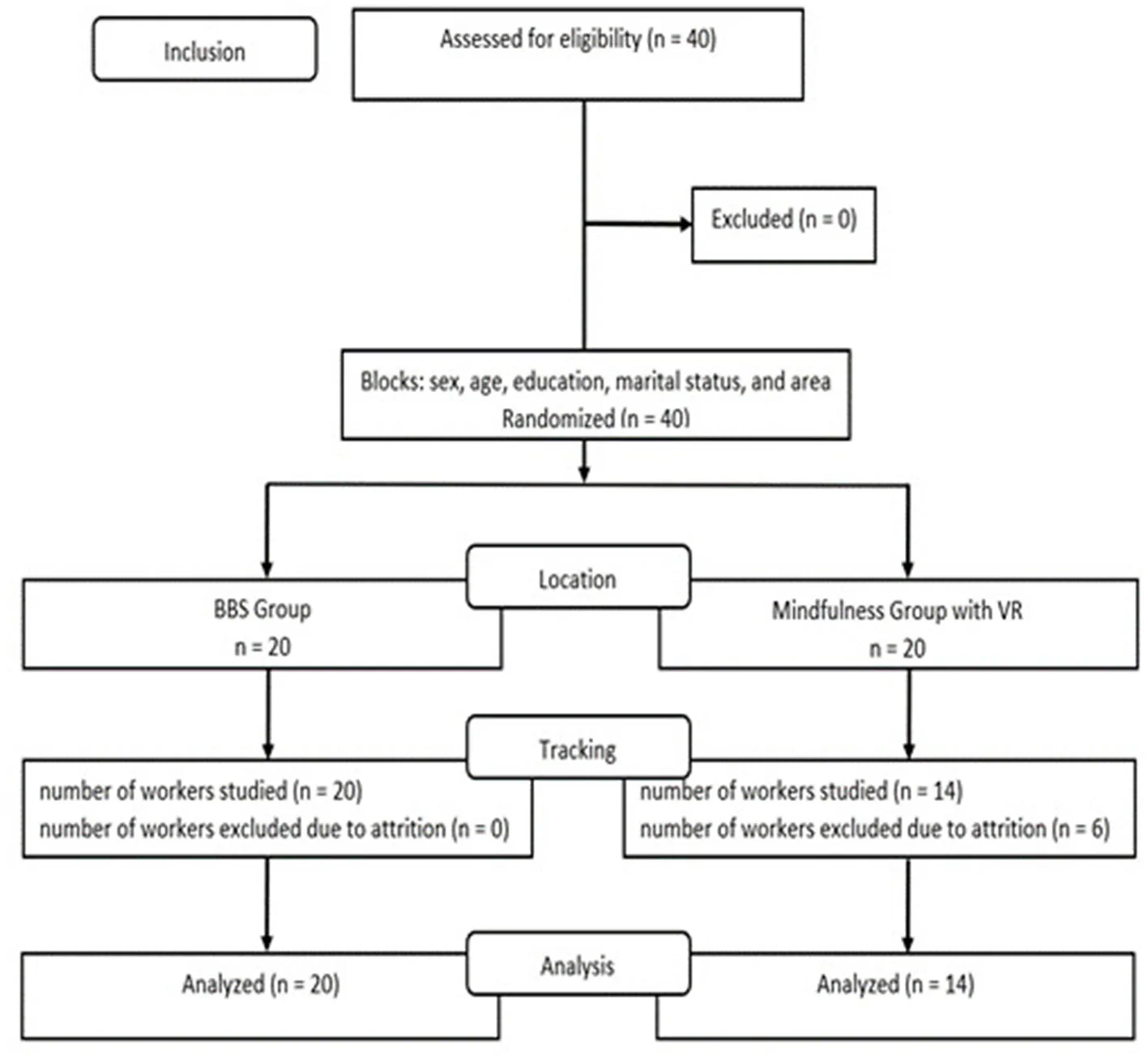
Consort flowchart of the sample.

**Table 1:** Sociodemographic characteristics of the experimental and control groups (*N* = 34).

Sociodemographic characteristics	BBS control group (*n* = 20)	Mindfulness group (*n* = 14)
Age M (SD)	39.7 (10.64)	43.0 (12.89)
Sex		
Female	4 (20%)	2 (14%)
Male	16 (80%)	12 (86%)
Level of education		
High school	12 (60%)	7 (50%)
Technician	8 (40%)	7 (50%)
Marital status		
Single	7 (35%)	4 (28.6%)
Married/cohabiting	13 (65%)	10 (71.4%)
Area		
Maintenance	2 (10%)	2 (14%)
Processes	3 (15%)	2 (14%)
Production	8 (40%)	7 (50%)
Supply chain	7 (35%)	3 (22%)

Due to the nature of the VR intervention, blinding participants to their treatment was not possible. However, the research hypothesis was masked so that participants were unaware of which program was considered the primary intervention. To reduce social desirability bias, sessions were held at different times. The study also employed a blind evaluation procedure; the sessions were led by a researcher who did not participate in the initial and subsequent evaluation, in order to be neutral and avoid any bias due to expectations.

Finally, group assignment ensured homogeneity in sample size and equivalence of participants' socioeconomic characteristics. During program implementation, six participants (30%) dropped out of the mindfulness group. The reasons reported were: pregnancy (*n* = 1), scheduling difficulties with the sessions (*n* = 3) and unspecified personal decision (*n* = 2). There were no dropouts in the SBC group. Data analysis was performed according to protocol, including only participants who completed the intervention in its entirety. This approach was chosen to evaluate the program's specific effectiveness under conditions of full session attendance. To minimize the impact of this differential dropout rate on internal validity, the initial equivalence of the groups was verified. As shown in Tables 2, 3, there were no significant differences in the pre-test measurements between the final groups, confirming the effectiveness of randomization in the initial allocation.

**Table 2:** Comparison between the control (BBS) and experimental (mindfulness) groups in terms of attitude toward risk prevention and its dimensions in the pre- and post-evaluation.

Subscale/group	*N*	Pre-test					Post-test				
		M	SD	*t*	*p*	g Hedges [95% CI]	M	SD	*t*	*p*	g Hedges [95% CI]
Confidence											
BBS control group	20	19.8	4.76	0.56	0.583	0.189 [−0.481; 0.856]	22.2	2.94	−1.03	0.309	−0.352 [−1.021; 0.323]
Mindfulness experimental G.	14	18.9	4.10				23.1	1.86			
Destiny											
BBS control group	20	15.3	3.21	1.45	0.157	0.493 [−0.189; 1.166]	14.9	1.18	−5.61	< 0.001	−1.909 [−2.712; −1.086]
Mindfulness experimental G.	14	13.6	3.72				17.6	1.65			
Participation											
BBS control group	20	20.1	3.80	1.21	0.237	0.410 [−0.267; 1.081]	21.7	2.39	−1.88	0.070	−0.638 [−1.318; 0.051]
Mindfulness experimental G.	14	18.4	4.62				23.0	1.47			
Avoidance											
BBS control group	20	23.9	4.80	1.52	0.137	0.518 [−0.164; 1.191]	23.3	1.22	−5.27	< 0.001	−1.794 [−2.581; −0.986]
Mindfulness experimental G.	14	21.1	5.50				27.4	3.20			
Belonging											
BBS control group	20	10.5	2.28	0.49	0.626	0.167 [−0.502; 0.834]	11.1	1.54	−0.79	0.438	−0.267 [−0.935; 0.405]
Mindfulness experimental G.	14	10.1	1.75				11.5	1.79			
Discomfort											
BBS control group	20	15.7	3.21	1.86	0.072	0.634 [−0.055; 1.314]	13.4	1.47	−3.54	0.001	−1.206 [−1.927; −0.469]
Mindfulness experimental G.	14	13.6	3.10				16.1	2.90			
Attitude toward risks p											
BBS control group	20	117.1	18.12	1.57	0.126	0.534 [−0.149; 1.209]	117.7	8.79	−4.1	< 0.001	−1.396 [−2.136; −0.638]
Mindfulness experimental G.	14	106.3	21.76				131.5	10.80			

M, mean; SD, standard deviation; *t*, Student's *t* test; *p, p* value; g, g of Hedges; CI, confidence interval.

**Table 3:** Comparison between the control (BBS) and experimental (mindfulness) groups in terms of work stress and its dimensions in the pre- and post-assessment.

Subscale/group	*N*	Pre-test					Post-test				
		M	SD	t	*p*	g Hedges [95% CI]	M	SD	t	*p*	g Hedges [95%CI]
Organizational climate											
BBS control group	20	12.0	3.84	−1.06	0.299	−0.359 [−1.029; 0.316]	15.3	2.36	5.45	< 0.001	1.853 [1.037; 2.648]
Mindfulness experimental G.	14	13.5	4.40				10.9	2.25			
Organizational structure											
BBS control group	20	13.0	4.75	0.34	0.739	0.114 [−0.554; 0.781]	14.9	2.56	1.96	0.059	0.667 [−0.024; 1.349]
Mindfulness experimental G.	14	12.4	5.47				13.1	2.99			
Organizational territory											
BBS control group	20	7.9	3.09	−1.79	0.083	−0.608 [−1.287; 0.080]	11.4	1.96	4.83	< 0.001	1.642 [0.855; 2.410]
Mindfulness experimental G.	14	10.0	3.74				8.6	1.01			
Technology											
BBS control group	20	9.3	3.19	−0.45	0.658	−0.152 [−0.819; 0.517]	11.4	2.30	2.04	0.051	0.692 [0.000; 1.375]
Mindfulness experimental G.	14	9.8	3.00				9.9	1.69			
Leading influence											
BBS control group	20	12.1	6.10	−0.26	0.793	−0.090 [−0.756; 0.578]	15.9	3.66	4.65	< 0.001	1.581 [0.801; 2.342]
Mindfulness experimental G.	14	12.6	4.94				10.9	1.96			
Lack of cohesion											
BBS control group	20	12.7	4.86	0.29	0.777	0.097 [−0.571; 0.764]	14.9	2.46	2.60	0.014	0.886 [0.179; 1.580]
Mindfulness experimental G.	14	12.2	4.89				11.9	4.24			
Group support											
BBS control group	20	9.0	4.14	−0.91	0.370	−0.309 [−0.978; 0.364]	11.5	2.52	4.42	< 0.001	1.503 [0.733; 2.255]
Mindfulness experimental G.	14	10.3	3.93				7.6	2.59			
Work stress											
BBS control group	20	75.9	24.84	−0.52	0.604	−0.178 [−0.845; 0.492]	95.2	15.45	4.91	< 0.001	1.670 [0.879; 2.442]
Mindfulness experimental G.	14	80.7	28.55				72.8	8.59			

M, mean; SD, standard deviation; *t*, Student's *t*; *p, p* value; g, g of Hedges; CI, confidence interval.

#### 2.1.2 Procedure

##### 2.1.2.1 Programs

A mindfulness-based program was developed, plus a control program, i.e., the Behavior-Based Safety model (BBS), which is the one used by this type of company. The program was implemented for 9 weeks. The meditation tasks contained in the program were also left as activities for each participant to complete at home. In these at-home activities, only support tasks were performed, but there were no records or evaluations of these tasks. One researcher led all sessions to control for inter-rater variability, and also received training in mindfulness and virtual reality management.

The mindfulness-based program incorporated techniques such as conscious breathing, guided imagery, and body scanning, supported by specific resources and materials for each session. Additionally, virtual reality equipment and software were employed during sessions 3, 4, and 6. The intervention consisted of nine sessions, each lasting 30 min. The sessions were structured as follows: motivation, exploration, and problematization (5 min); knowledge construction (10 min); transfer (10 min); evaluation (3 min); and closure (2 min). The minutes of VR exposure in sessions 3, 4 and 6 were 10 min in each session, and corresponded to the transfer part, therefore if we add the 10 min of each session, we would have 30 min of total VR exposure time in the entire program that each participant had.

Session 1 introduced the concept of mindfulness, fostering interest in its theoretical and practical aspects. Session 2 focused on guided body visualization and exploration, aiming to understand its principles and applications. In session 3, virtual reality was introduced to facilitate relaxation and observation of stressors, with the goal of raising awareness about the effects of stress and practicing strategies for its management. Session 4 continued with virtual reality and emphasized “body exploration and active pause” to explore the practical relationship between active pauses and mindfulness. Session 5 centered on the application of mindfulness to thought processes and walking meditation, aiming to understand its benefits and connections to previous techniques (VR was not used in this session). Session 6, the final VR-based session, focused on observing stressors and their physiological correlates, accompanied by relaxation exercises. Session 7 addressed seated meditation and guided body scanning. In session 8, the participants worked on attention and fluency in work-related tasks. Finally, session 9 focused on the integration of mindfulness into daily life and drew the program to a close.

The intervention was delivered by a single facilitator, a licensed mental health professional certified in mindfulness-based. The facilitator served as the lead instructor for all sessions and completed 40 h of formal training and supervised practice in mindfulness-based approaches, including specific training in the therapeutic application of virtual reality. No additional facilitators conducted sessions; however, one member of the research team provided logistical support and observational monitoring without direct involvement in session delivery.

To ensure implementation fidelity, a structured session-by-session checklist based on the intervention protocol was systematically completed by the facilitator after each session. Fidelity monitoring was independently conducted by two members of the research team, who supervised some sessions. Inter-rater agreement exceeded 85%, indicating high concordance and satisfactory levels of adherence and reliability in program implementation.

This mindfulness program incorporates several design features that distinguish it from conventional face-to-face formats. First, it integrates traditional mindfulness techniques with virtual reality (VR) technology, providing participants with an immersive environment intended to support attentional engagement and experiential learning (6). Prior literature suggests that immersive and technology-assisted mindfulness interventions may enhance engagement and facilitate stress management processes.

Second, the program emphasizes practical application in everyday work contexts. Sessions explicitly address stress management in occupational settings and the development of mindfulness skills that may be transferred to daily professional activities (1). This applied focus aligns with approaches that promote the integration of mindfulness practices into real-life professional demands.

Third, the intervention follows a structured format with ongoing assessment. Participants are guided progressively through mindfulness practices, with opportunities for reflection and structured feedback. Continuous monitoring procedures are incorporated to support skill consolidation and adherence to the protocol (19). Structured implementation and evaluation frameworks have been recommended to enhance consistency and facilitate outcome assessment.

Overall, rather than positioning the program as uniquely innovative, it may be more appropriately described as a structured, technology-assisted adaptation of mindfulness-based practice tailored to occupational stress contexts. Its integration of immersive technology within a workplace-oriented framework represents a contextualized extension of existing mindfulness approaches (25), the effectiveness of which requires continued empirical evaluation.

##### 2.1.2.2 Virtual reality (VR) programs

To create an immersive environment conducive to practicing meditation in a simulated natural context, the virtual reality intervention was delivered using Oculus Quest 2 standalone head-mounted displays (Meta Inc.), without connection to an external computer or tracking stations. The software application Nature Treks VR (Version 2.15) was run natively on the device. The system operated at a refresh rate of 72 Hz, with an approximate field of view of 90 ° and a display resolution of 1,832 × 1,920 pixels per eye, consistent with the manufacturer's specifications.

The VR component was implemented in three specific sessions (sessions 3, 4, and 6), with an exposure duration of approximately 10 min per session, resulting in a total of 30 min of VR immersion per participant across the program. Prior to their first VR session, participants completed a brief 3–5-min familiarization phase to acclimate to the headset and reduce the risk of cybersickness.

During the VR experience, participants remained seated and were instructed to maintain a relaxed posture with minimal head movement. Inside-out head tracking provided by the Oculus Quest 2 was enabled, allowing for natural, slow head movements, while no locomotion or manual interaction was required, thereby minimizing sensory conflict. The selected virtual environment, Green Meadows, featured dynamic natural scenery, ambient sounds (e.g., wind, birds, and running water), and light visual interactivity, designed to support relaxation and focused attention without excessive sensory stimulation.

All sessions were conducted in a controlled, quiet room free of external distractions. Before each session, technical checks of the VR headsets were performed, and facilitators provided standardized usage instructions and assisted with headset placement to ensure comfort and safety. Hygiene protocols were strictly followed, including visor cleaning between participants. Environmental conditions such as lighting, temperature, and noise levels were monitored to maintain consistency across sessions.

Cybersickness symptoms (e.g., nausea, dizziness, or visual discomfort) were monitored informally through post-session check-ins. No severe adverse events were reported, and minor symptoms (e.g., transient eye fatigue or slight disorientation) were rare. Participants were informed that they could withdraw from the VR component at any time if discomfort occurred; however, none chose to do so. To prevent contamination between conditions, control and experimental group sessions were scheduled at different times and conducted in separate physical spaces. Additionally, evaluators responsible for pre- and post-intervention assessments were blinded to group allocation, minimizing potential bias in data collection.

##### 2.1.2.3 Virtual reality equipment

Considering the growing adoption of virtual reality (VR) applications for mindfulness, it is crucial to address the technical characteristics of VR equipment and software, as variations in resolution, refresh rate, tracking precision, and content quality can significantly influence the user's immersive experience and, consequently, the effectiveness of VR-based interventions (42).

For this study, we used the latest-generation HTC VIVE Cosmos Elite VR system (VIVE, HTC Corporation), a device recognized for its high-fidelity immersive capabilities and accurate spatial tracking. The headset features a combined resolution of 2,880 × 1,700 pixels (1,440 × 1,700 pixels per eye), a refresh rate of 90 Hz, and a field of view of approximately 110 °, ensuring smooth visual rendering and minimizing motion latency. The system supports IPD (interpupillary distance) adjustment ranging from 58 to 72 mm, allowing customization for different users in order to optimize visual comfort and reduce visual strain.

In the tracking configuration, we employed VIVE Base Station 2.0 sensors, positioned diagonally at a height of approximately 2 meters and separated by a distance of 4.5 meters, providing accurate six-degree-of-freedom (6DoF) motion tracking within a defined 2.5 × 2.5-meter interaction area. The setup was calibrated before each session to ensure alignment and prevent tracking drift. All sessions were conducted using the same spatial configuration and lighting conditions to guarantee consistency across participants.

The system was operated using a high-performance gaming laptop equipped with an NVIDIA RTX 3060 GPU, 16 GB of RAM, and an Intel i7 processor, capable of maintaining a stable framerate throughout the experience. The VR content was delivered via the STEAM VR and VIVEPORT platforms, ensuring standardized software deployment. All necessary accessories—VR controllers, base stations, cables, power adapters, and chargers—were installed according to the manufacturers' specifications. This configuration provided a stable, high-fidelity virtual environment suitable for immersive mindfulness experiences and ensured the experimental conditions were reproducible.

##### 2.1.2.4 Procedure

The study received ethical approval from both the University's Ethics Committee and the Company's Management. Prior to the implementation of the program, an invitation letter was sent, and three preparatory meetings were held to coordinate logistical aspects and collect employee data for group allocation. During these sessions, written informed consent was obtained, ensuring the participants' involvement was voluntary and confidential.

Data collection was conducted at two time points: pre-intervention (baseline) and post-intervention (after 9 weeks). Given the variability in work schedules, each intervention session (excluding the control group) was offered twice weekly—with morning sessions on Mondays, Tuesdays, and Wednesdays and evening sessions on Thursdays, Fridays, and Saturdays—to accommodate different shifts. Both the intervention and control conditions were implemented simultaneously over a 9-week period.

To maintain methodological rigor, several measures were employed to minimize bias and contamination. Outcome assessors were blinded with respect to the groups to which the participants were assigned to prevent expectancy effects during evaluation. The facilitator of the intervention did not collect the data, which ensured the independence of the procedure. To prevent cross-group contamination or cointervention, sessions for the experimental and control groups were held in separate physical spaces and scheduled at different times. The participants were instructed not to discuss session content with colleagues from other groups, and this separation was reinforced throughout the study. Communication between groups was monitored informally by supervisors to confirm adherence to these procedures.

All assessment instruments were administered in person to promote understanding and reduce response errors. This person was a blind evaluator at the beginning and end; he did not know who was in the control group and who was in the experimental group. The participants were reminded that their responses would remain confidential and that there were no “right” or “wrong” answers. This approach was intended to foster honest feedback and ensure the reliability and validity of the collected data.

##### 2.1.2.5 Measurement

Three assessment tools were used in this study.

To evaluate the participants' attitudes toward safety, the scale of attitudes toward the prevention of occupational risks and accidents (PRP), developed by Luis López Mena in its Spanish version, was used. This comprehensive evaluation consisted of 30 items, which were organized into six different factors: trust (items 23, 24, 25, 26, and 29), destiny (items 4, 6, 7, and 27), participation (items 1, 2, 11, 15, and 24), avoidance (items 3, 9, 12, 13, 20, and 22), belonging (items 18, 19, and 21), and discomfort (items 5, 10, 16, and 30). The final score was obtained by adding the answers to the 30 items.

This scale presents participants with statements or phrases to measure their agreement or disagreement on a 5-point scale, ranging from “Strongly agree” to “Strongly disagree,” and certain items are scored inversely. Originating in Spain in 1977, the scale was adapted and standardized by Chile's National Security Council, thereby maintaining its reputation as a reliable measure of security attitudes. Validation studies yielded an acceptable reliability coefficient of 0.74, as determined using the split-half method. The factor structure of the scale, composed of six factors and 30 items, includes complementary items for the preparation of the form.

A validation study involving 161 workers over the course of a year revealed a significant correlation (0.41) between PRP scores and accident frequency, suggesting that lower PRP scores may indicate greater susceptibility to accidents among workers. In addition, empirical validity studies highlighted correlations between the total score and individual factors—confidence (0.14), destiny (0.75), avoidance (0.60), participation (0.60), belonging (0.67), and discomfort (0.96)—emphasizing the effectiveness of the scale in identifying potentially accident-prone individuals and assessing various safety attitudes among workers (41).

The ILO-WHO Work-Related Stress Questionnaire served as a tool for assessing stress levels among workers. Widely adopted in organizations, this self-report measure consists of 25 items distributed across seven factors: organizational climate (items 1, 10, 11, and 20), organizational structure (items 2, 12, 16, and 24), organizational territory (items 3, 15, and 22), technology (items 4, 14, and 25), leader influence (items 5, 6, 13, and 17), lack of cohesion (items 7, 9, 18, and 21), and group support (points 8, 19, and 23). Respondents rate each item on a scale of 1 to 7, indicating the degree to which they perceive each condition as a source of stress, ranging from “never” (1) to “always” (7).

The validity of the questionnaire was established in the Mexican population using the Kaiser–Meyer–Olkin (KMO) measure, yielding a value of 0.915, and Bartlett's significant sphericity test (*p* < 0.000). Confirmatory factor analysis revealed two factors: “Organizational Conditions”, which includes 21 items related to the structure and cohesion of and support for the work groups, and “Administrative Processes”, which consists of 4 items related to administrative procedures within the organization. Both factors were found to have relevant validity scores of 65%. The score for the reliability of the instrument, assessed using Cronbach's alpha method, yielded a score of 0.92, indicating high suitability for the study population (31).

##### 2.1.2.6 Data analysis

To detect differences between the two groups in terms of the self-reported measures of the two dependent variables and their dimensions, a Multivariate Analysis of Variance (MANOVA) was used as the primary analysis, assuming there was a linear relationship between the dependent variables. A partial eta-squared value and observed power (the probability of avoiding a Type II error) are also reported, as well as the Bonferroni correction for multiplicity control. In addition to this analysis, several secondary analyses were performed, including a comparison of means between the two groups using an independent-samples *t*-test and a pre-test/post-test comparison between the two groups using a paired-samples *t*-test. The assumptions were verified for the analysis; for normality, the Shapiro–Wilk test (*p* ≥ 0.05) was used for both groups; for homogeneity of variances, Levene's test (*p* > 0.05) was used (32). The means of the two groups were compared in the pre-test, and no significant differences were found in the dependent variables, i.e., attitude toward risk prevention and work-related stress. This would demonstrate an initial equivalence between the control and experimental groups. To assess the magnitude of the program's results, we calculated the effect size using Hedges' g and its 95% confidence intervals. Values between 0.20 and 0.40 were considered small effect sizes, values between 0.5 and 0.7 were considered moderate effect sizes, and values greater than 0.8 were considered large effect sizes (33–35). To analyze the effect in each group, we used the paired Student's *t*-test to observe the trend in scores as well as Cohen's d to assess the effect size and its 95% confidence intervals.

## 3 Results

A Multivariate Analysis of Variance (MANOVA) was performed due to the presence of two dependent variables and their respective dimensions. Pillai's Trace was used as the test statistic because of its robustness; it is employed when covariance matrices cannot be interpreted as equal. The analysis showed significant overall differences between the groups [V = 0.81, *F*(14, 19) = 5.86, *p* < 0.01], with a partial eta-squared effect size (“η”–*p*^∧^2) of 0.812, indicating a strong relationship (>0.7), and an observed power of 0.995, representing the probability of avoiding a Type II error (Table 4). After verifying the assumption of homogeneity of variances (*p* > 0.05), the Bonferroni correction was applied to pairwise comparisons to control the family work error rate (FWER). Two families of tests were defined: (1) attitudes toward prevention (k = 7), with an adjusted significance level of α = 0.007; and (2) job stress (k = 8), with an adjusted level of α = 0.006. Table 5 presents the raw *p*-values, which were compared with these adjusted thresholds to determine significance.

**Table 4:** Results of the multivariate tests (MANOVA) for the variable attitude toward accident prevention and its dimensions and work stress and its dimensions.

Source	Statistical	Valor	*F*	gl (hypothesis)	gl (error)	*p*	$\eta_{p}^{2}$
Intervention	Pillai's trace	0.81	5.864	14.000	19.000	< 0.01	0.812

F, The F-statistic value; gl, Degrees of freedom; gl (error), Degrees of freedom for the error or residuals; *p*, the *p*-value; $\eta_{p}^{2}$, Partial eta squared.

**Table 5:** Results of the multivariate tests (MANOVA) using Bonferroni's correction for attitude toward accident prevention with its dimensions and occupational stress and its dimensions.

Dependent variable	Mean difference	Standard error	Unadjusted *p*	Adjusted α	Decision	95% CI difference [L. L., U. L.]
Confidence	1.793	1.923	0.358	0.007	Not significant	[−2.124, 5.709]
Destiny	4.450^*^	1.351	0.002	0.007	Significant	[1.698, 7.202]
Participation	3.093	1.599	0.062	0.007	Not significant	[−0.165, 6.351]
Avoidance	6.836^*^	1.808	0.001	0.007	Significant	[3.152, 10.519]
Belonging	0.807	0.966	0.410	0.007	Not significant	[−1.162, 2.776]
Discomfort	4.729^*^	1.073	0.000	0.007	Significant	[2.542, 6.915]
Attitudes toward risk prevention	24.564^*^	7.013	0.001	0.007	Significant	[10.279, 38.849]
Organizational climate	−5.893^*^	1.485	0.000	0.006	Significant	[−8.917, −2.868]
Organizational structure	−1.286	1.761	0.471	0.006	Not significant	[−4.872, 2.300]
Organization territory	−4.857^*^	1.254	0.000	0.006	Significant	[−7.411, −2.303]
Technology	−1.957	1.259	0.130	0.006	Not significant	[−4.521, 0.607]
Leading influence	−5.514	2.238	0.019	0.006	Not significant	[−10.073, −0.955]
Lack of cohesion	−2.507	1.934	0.204	0.006	Not significant	[−6.447, 1.432]
Group support	−5.214^*^	1.707	0.005	0.006	Significant	[−8.691, −1.737]
Work stress	−27.229	9.631	0.008	0.006	Not significant	[−46.847, −7.610]

CI difference, 95% confidence interval; LL, lower limit, UL, upper limit; this table presents pairwise comparisons between groups. Unadjusted *p* values are compared against the adjusted α (Bonferroni) to determine significance. For Attitudes, *k* = 7 (α_*adj*_= 0.007); for Work Stress, *k* = 8 (α_*adj*_= 0.006). ^*^Significant at the adjusted level.

Student's *t*-tests were also used to analyze the differences between the two groups (control and mindfulness) in terms of attitude toward risk prevention and its dimensions (trust, fate, participation, avoidance, belonging, and discomfort) as well as work-related stress and its dimensions (organizational climate, organizational structure and territory, technology, leader influence, lack of cohesion, and group support), with 95% confidence intervals. The results in Table 2 show that there were no significant differences in the initial assessment of attitude toward risk prevention. In the post-test comparison of both groups, there were statistically significant differences in “fate,” “avoidance,” and “discomfort,” with significantly increased means, especially in the experimental group (mindfulness), and with acceptable effect sizes. The mindfulness program led to improvements in “destiny” because by improving awareness, a worker rejects beliefs, myths, and superstitions formed in an attempt to explain workplace accidents; they instead attribute the accident to human behavior related to accident prevention. It also improved “avoidance” because the workers developed personal, family, and professional commitments and responsibility regarding tasks and actions that reduce the incidence of workplace accidents. Similarly, by explaining the causes of these accidents, the same occurs with “discomfort,” as workers actively incorporate tasks and practices aimed at preventing workplace accidents, considering these tasks as necessary and as a condition that facilitates their work (Table 2).

In the intragroup analysis, that is, the pre- and post-tests performed using the paired-groups Student's *t*-test, the control group (BBS) showed improvements in terms of means, but these were not statistically significant, while the mindfulness group achieved significant improvements in their means and with high effect sizes. Although there was a wide range in some intervals, since the values did not reach 0, this result indicates statistical significance, meaning that there is sufficient evidence to reject the null hypothesis with the established confidence level, except with respect to “belonging.” This may be because from the beginning, the workers assumed that tasks related to accident prevention are their individual responsibilities as part of their job duties and could not be delegated to others, so they did not wish to promote or incentivize others to do so. Regarding work-related stress and its dimensions (climate, organizational structure and territory, technology, leader influence, lack of cohesion, and group support), Table 3 shows that there were also no significant differences in the initial assessment between the two groups. In the post-test, we did find significant differences between both groups in terms of “job stress,” “organizational climate,” “organizational territory,” “leader influence,” and “group support.” However, the MANOVA results indicated that only “organizational climate,” “organizational territory,” and “group support” were significant. These results suggest that, in the case of “job stress,” by reducing emotional reactivity, employees can better manage stressful situations without feeling overwhelmed. This allows them to reduce anxiety and handle job demands more effectively, leading to a decrease in perceived stress (39). In the case of “organizational climate,” this situation has occurred because mindfulness, in the organizational context, has been linked to greater self-awareness, reduced stress, better emotional management, and increased empathy and collaboration in the workplace (Hofmann & Gómez, 2017). Third, regarding the “organizational territory” factor, mindfulness enabled employees to become more aware of their emotions, thoughts, and behaviors, which can lead to a greater sense of self-control and autonomy within their work environments (36). Fourth, concerning “leader influence,” mindfulness is known for its ability to improve emotional self-regulation, which is especially important for leaders. Practicing mindfulness makes employees better able to manage stress, frustration, and tension in difficult situations without these emotions negatively impacting their decision-making or their behavior toward the team (36). Fifth, concerning “group support,” mindfulness fosters greater awareness of the emotions, thoughts, and needs of others (37). This increased awareness can lead workers to be more empathetic and willing to offer support when their colleagues need it. When team members are more aware of each other's emotions and needs, they can offer greater emotional and practical support (38). It should also be noted that the results show high effect sizes > 0.8 (Table 3).

Regarding the within-group comparison, that is, the pre- and post-test results, we can see that job stress and its dimensions tended to increase in the control group (BBS), while they decreased in the experimental group (mindfulness). However, this difference is only significant in the “organizational climate” dimension, with a medium effect size of 0.624; the effect sizes for the other dimensions are greater than 0.8 (Table 6).

**Table 6:** Intragroup comparison (pre- and post-test) of the control group (BBS) and the experimental group (mindfulness) in terms of attitudes toward safety and work stress.

Dependent variable/dimensions	BBS control group						Mindfulness experimental group					
	Pre-test		Post-test		*p*	d [95% CI]	Pre-test		Post-test		*p*	d [95% CI]
	M	SD	M	SD			M	SD	M	SD		
Confidence	19.8	4.76	22.2	2.94	0.115	−0.369 [−0.819; 0.089]	18.9	3.96	23.1	1.86	0.002	−1.041 [−1.686; −0.372]
Destiny	15.3	3.21	14.9	1.18	0.577	0.127 [−0.315; 0.565]	13.6	3.58	17.6	1.65	0.004	−0.927 [−1.546; −0.283]
Participation	20.1	3.80	21.7	2.39	0.179	−0.312 [−0.757; 0.141]	17.9	4.74	23.0	1.47	< 0.000	−1.169 [−1.843; −0.469]
Avoidance	23.9	4.80	23.3	1.22	0.609	0.116 [−0.325; 0.555]	21.1	5.30	27.4	3.20	0.001	−1.084 [−1.738; −0.405]
Belonging	10.5	2.28	11.1	1.54	0.422	−0.183 [−0.623; 0.261]	9.7	2.31	11.5	1.79	0.055	−0.564 [−1.121; 0.011]
Discomfort	15.7	3.21	13.4	1.47	0.004	0.735 [0.231; 1.223]	13.5	3.02	16.1	2.90	0.010	−0.808 [−1.404; −0.189]
Attitudes toward risk prevention	117.1	18.12	117.7	8.79	0.892	−0.031 [−0.469; 0.408]	105.3	21.33	131.5	10.80	< 0.000	−1.354 [−2.075; −0.606]
Organizational climate	12.0	3.84	15.3	2.36	0.003	−0.760 [−1.252; −0.252]	13.5	4.24	10.9	2.25	0.036	0.624 [0.390; 1.189]
Organizational structure	12.9	4.75	14.9	2.56	0.084	−0.408 [−0.860; 0.054]	12.5	5.29	13.1	2.99	0.620	−0.136 [−0.659; 0.393]
Organization territory	7.9	3.09	11.4	1.96	< 0.000	−0.995 [−1.526; −0.447]	10.1	3.64	8.6	1.01	0.195	0.366 [−0.178; 0.901]
Technology	9.3	3.19	11.4	2.30	0.015	−0.601 [−1.072; −0.117]	9.9	2.90	9.9	1.69	0.890	−0.038 [−0.561; 0.487]
Leading influence	12.1	6.10	15.9	3.66	0.026	−0.539 [−1.003; −0.063]	12.5	4.77	10.9	1.96	0.255	0.318 [−0.225; 0.850]
Lack of cohesion	12.7	4.86	14.9	2.46	0.068	−0.432 [−0.886; 0.032]	12.6	4.94	11.9	4.24	0.835	0.057 [−0.469; 0.580]
Group support	9.0	4.14	11.5	2.52	0.035	−0.508 [−0.968; −0.035]	10.7	4.17	7.6	2.59	0.057	0.559 [−0.016; 1.115]
Work stress	75.9	24.84	95.2	15.45	0.005	−0.708 [−1.192; −0.208]	81.8	27.83	72.8	8.59	0.311	0.281 [−0.258; 0.811]

M, Mean; SD, standard deviation; *p*, value of *p*; d, Cohen's D; CI, confidence interval.

The effectiveness of the program is more immediately evident in the graphs, as they make it clear that job stress decreased in the group subjected to the mindfulness program, while it remained the same in the control group (Figures 2, 3).

**Figure 2:**
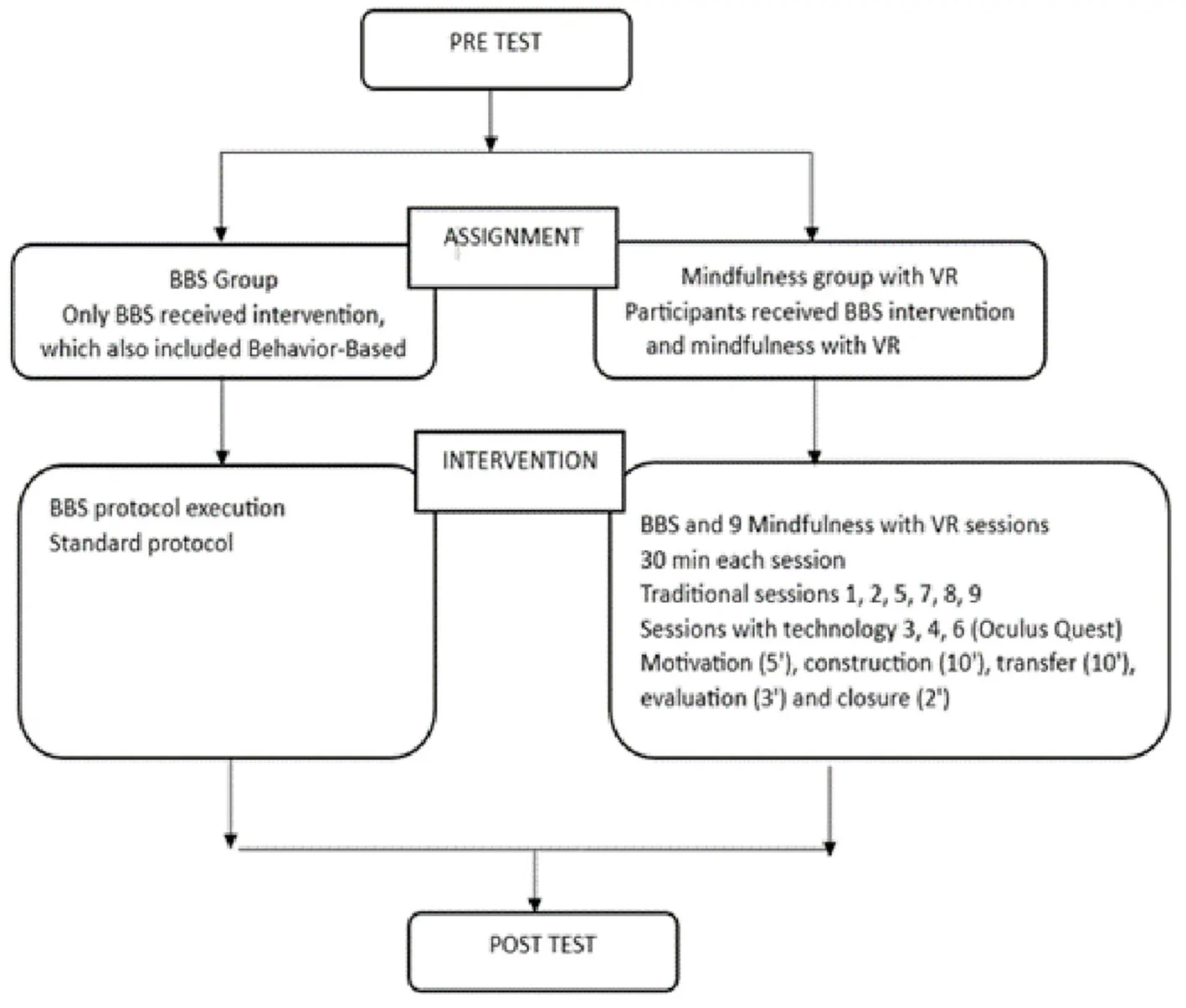
Consort flowchart of procedures.

**Figure 3:**
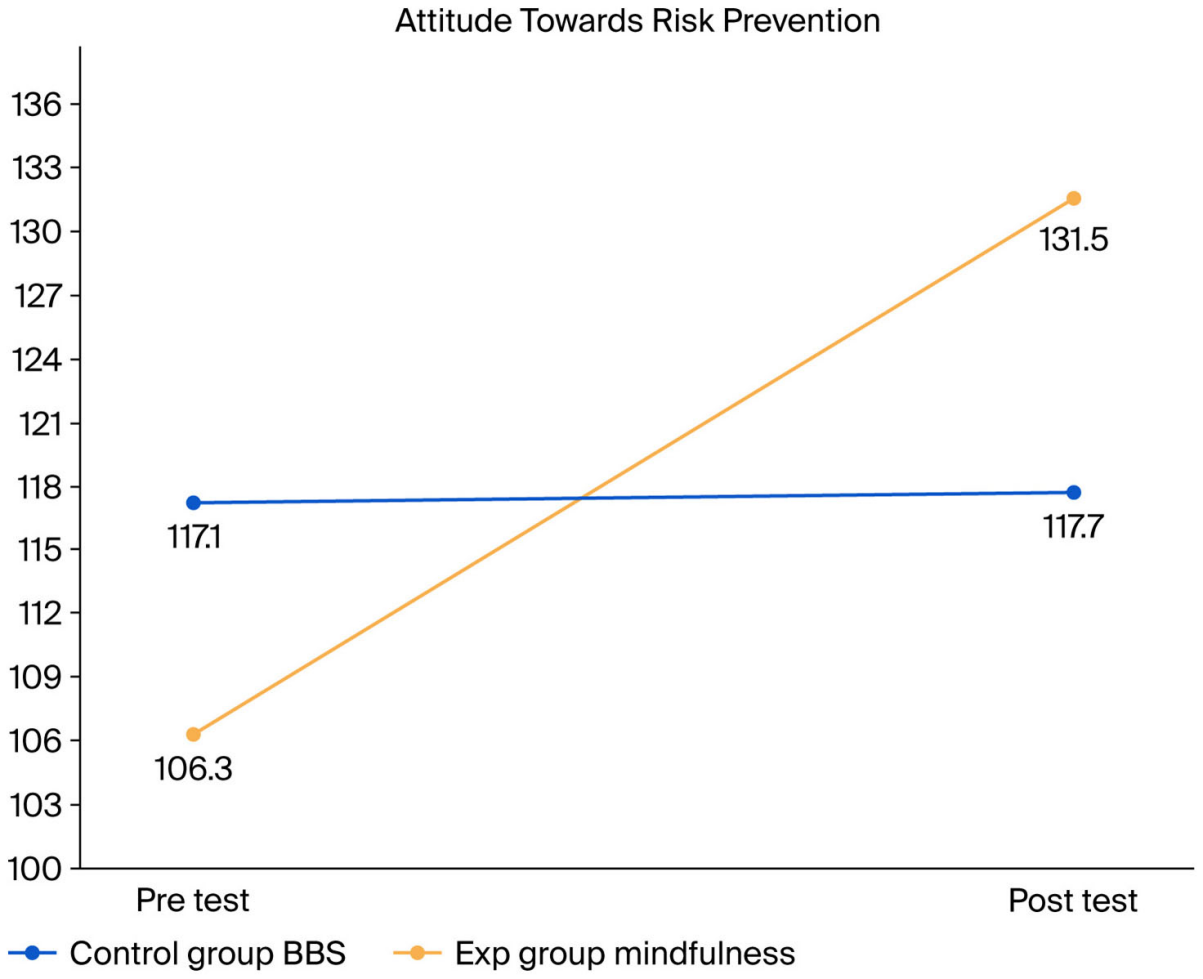
Comparison of the control group (BBS) and the experimental group (mindfulness) in terms of attitudes toward risk prevention.

**Figure 4:**
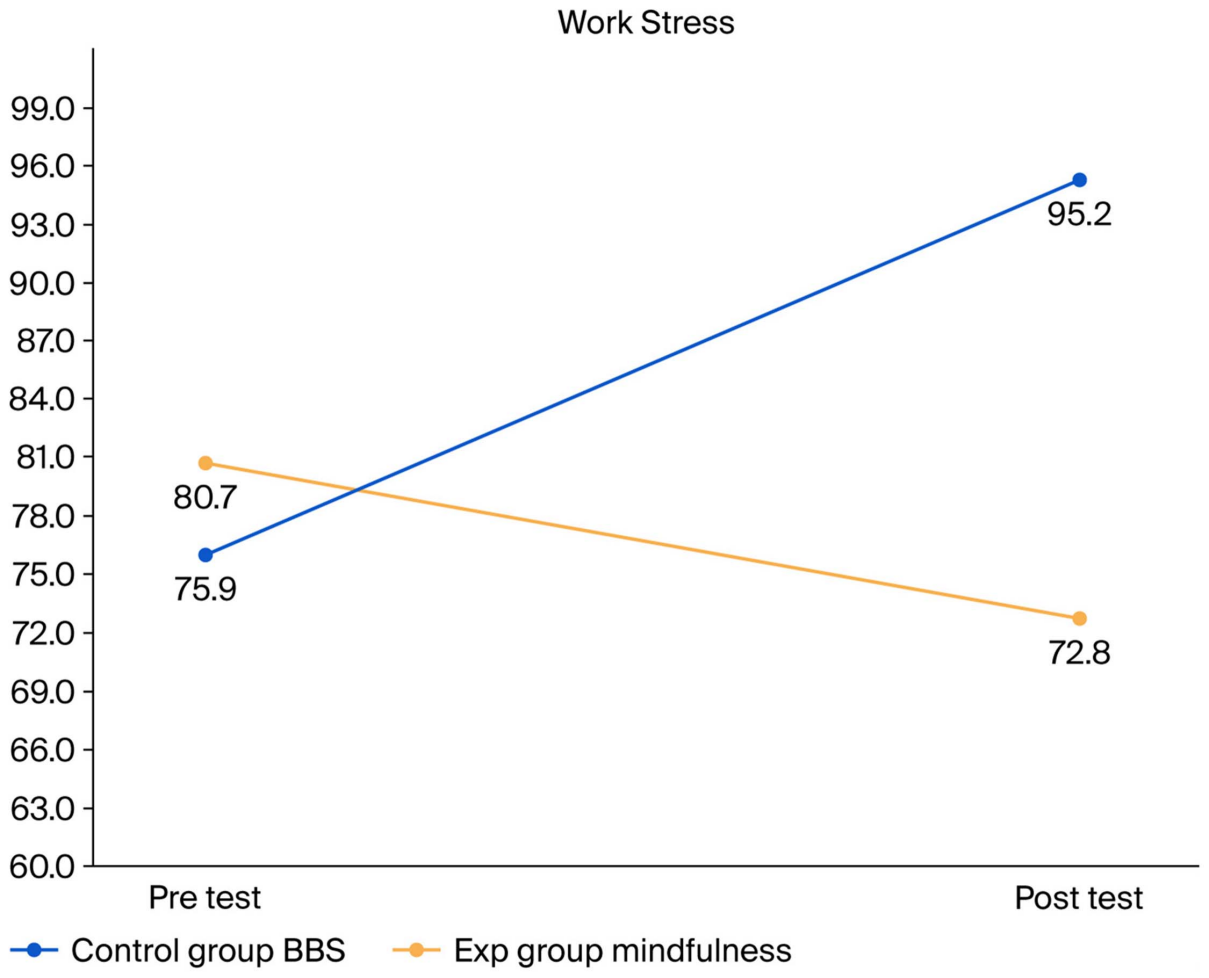
Comparison of the control group (BBS) and the experimental group (mindfulness) in terms of work stress.

## 4 Discussion

Evidence indicates that mindfulness is a valuable tool for improving attitudes toward occupational risk prevention across all its dimensions. This is reflected in the present study, where its impact on both risk prevention attitudes and work-related stress was examined. The findings demonstrate that attitudes toward risk prevention—and their underlying dimensions—improved significantly following the mindfulness-based program. This is consistent with the findings of Valley and Stallones (23), who reported that mindfulness reduces cognitive failures in the workplace and strengthens safety behaviors, ultimately contributing to a reduction in workplace accidents (25).

Specifically, a notable increase was observed in the “destiny” factor, suggesting that workers became less likely to endorse beliefs, myths, or superstitions as explanations for workplace accidents. Instead, accidents were more frequently attributed to human behavior and preventive actions (22). A substantial increase was also observed in “avoidance,” indicating greater recognition of personal, family, and occupational responsibilities associated with behaviors that reduce accident occurrence, including when explaining their causes (20). Similarly, improvements were found in “discomfort,” reflecting a greater tendency among workers to avoid behaviors that may lead to workplace accidents (17).

Regarding work stress and job satisfaction, the results indicate that the program had a significant effect on both variables, contributing to reduced stress levels and increased job satisfaction. These findings suggest that mindfulness helps workers develop greater awareness of their values and needs, enhancing job satisfaction through improved emotional regulation (17).

The observed results are unlikely to be due to chance, as significant effect sizes were obtained for most variables, with the exception of “organizational structure” and “lack of cohesion.” This exception may be explained by insufficient development of interpersonal connections or organizational alignment among participants across groups (17).

The mindfulness program also produced positive effects in reducing work stress across several dimensions, including “organizational climate,” “organizational territory,” “leader influence,” and “group support.” These findings suggest that mindfulness, by fostering self-awareness and emotional regulation, contributes to improvements in employees' perceptions of their work environment.

In terms of organizational climate, mindfulness appears to promote a more positive and harmonious environment by enhancing emotional regulation and interpersonal awareness. Employees become more capable of managing their emotions and engaging in empathetic and collaborative relationships, which positively influences workplace interactions and overall climate (37, 38).

Regarding organizational territory—the physical and social space in which employees interact—the results indicate a significant reduction in negative perceptions. Mindfulness enhances awareness of thoughts, emotions, and behaviors, promoting a sense of autonomy and self-regulation. This, in turn, leads to more positive perceptions of the work environment and improved relationships within it (36, 39).

Leadership was also positively influenced. Leaders who practice mindfulness demonstrate greater emotional self-regulation, enabling them to manage stress and conflict more effectively without compromising decision-making. This contributes to a more stable and predictable work environment, strengthening employees' trust and reinforcing leadership credibility (36, 39).

Similarly, group support showed improvement. Mindfulness fosters empathy and awareness of others' needs, enhancing employees' capacity to provide emotional and practical support. This leads to stronger interpersonal relationships and a more collaborative and supportive work environment (37, 38).

Finally, a significant reduction in perceived work-related stress was observed. Reduced emotional reactivity allows employees to cope more effectively with job demands, decreasing anxiety and improving performance under pressure (39). This highlights the potential of mindfulness as an effective tool for stress management in organizational contexts.

Overall, the findings suggest that mindfulness interventions can improve multiple aspects of the work environment, contributing to both individual wellbeing and the development of healthier, more productive organizations. These results are consistent with previous research highlighting the benefits of mindfulness in reducing stress and improving workplace relationships (36, 37).

In contrast, the control group, which underwent the Behavior-Based Safety (BBS) program, showed an increase in work stress levels. This may be attributed to the cognitive and emotional demands associated with adapting to new behavioral expectations and responsibilities. The introduction of new competencies can increase cognitive load and generate anxiety, particularly when employees perceive insufficient resources or time to adapt (38).

Additionally, uncertainty regarding the program's effectiveness or implementation may have contributed to increased stress levels. A lack of clarity or support during organizational change processes can generate insecurity, making adaptation more difficult. The increase in stress observed in the post-test may therefore reflect the challenges associated with adjusting to new work demands, a phenomenon well documented in the literature on organizational change (7).

These findings suggest that, although BBS programs aim to improve safety and wellbeing, their implementation may initially increase stress if not accompanied by adequate support mechanisms. This highlights the importance of carefully managing transitions and providing sufficient guidance during implementation.

It is important to note that the multivariate analysis (MANOVA) indicated that only certain stress-related factors showed statistically significant differences, suggesting that these results should be interpreted with caution.

Furthermore, the findings indicate that mindfulness did not completely eliminate work-related stress but rather helped regulate and stabilize it. This may be explained by employees' adaptation to chronic stress conditions. In high-demand environments, workers may develop tolerance to stressors such as workload, interpersonal conflict, or job insecurity, limiting the extent to which mindfulness can reduce overall stress levels (37).

Instead, mindfulness appears to function as a regulatory mechanism, helping individuals manage stress intensity and maintain emotional balance rather than eliminating stressors entirely. Structural and organizational factors often remain unchanged, meaning that individual-level interventions alone may not fully address the root causes of stress (36).

Therefore, mindfulness should be understood as a complementary strategy for stress management, rather than a standalone solution. Its effectiveness may be enhanced when combined with organizational interventions aimed at reducing structural stressors.

The results of this study support the conclusion that mindfulness-based programs significantly improve employees' attitudes toward occupational risk prevention. The differences observed between the experimental and control groups support the study's main hypothesis and are consistent with previous research demonstrating positive relationships between mindfulness, safety attitudes, and reduced stress (18, 22, 23, 25).

Studies by Liang (18) and Molek and Żołnierczyk (22), conducted in high-risk sectors such as construction and mining, reported similar findings, showing that mindfulness-based interventions improve safety outcomes and reduce violations. However, Liang (18) also found that very high levels of mindfulness may be associated with increased autonomy and occasional noncompliance with safety procedures.

The findings of the present study are broadly consistent with previous research suggesting that mindfulness may contribute to occupational safety by improving attention, situational awareness, emotional regulation, and safety-related behaviors. An integrative review of 32 empirical studies concluded that mindfulness may facilitate the detection of workplace hazards, enhance concentration and self-monitoring of safety behaviors, and support more adaptive responses to emotional demands in safety-critical work environments (48). Similarly, previous experimental evidence from a high-risk mining context found that a mindfulness-based program supported by virtual reality produced a significant improvement in safe behavior compared with Behavior-Based Safety (BBS) alone, providing preliminary evidence for the integration of mindfulness and immersive technologies into occupational safety programs. These findings are compatible with the significant improvement in attitudes toward occupational risk and accident prevention observed in the present study and suggest that mindfulness may strengthen safety-related outcomes by addressing psychological processes that are not directly targeted by conventional behavioral approaches.

However, the findings concerning work-related stress are less conclusive. Previous meta-analyses have generally reported beneficial effects of workplace mindfulness interventions on stress (43), but substantial heterogeneity across interventions, populations, and outcomes has also been reported. In addition, evidence from other occupational populations has been inconsistent; for example, a systematic review of mindfulness interventions among nurses found a reduction in stress but rated the certainty of the evidence as very low, highlighting the need for further high-quality studies. Therefore, the non-significant reduction in work-related stress observed in the present study may reflect differences in occupational context, intervention characteristics, and sample size rather than an absence of potential benefits of mindfulness. Taken together, these findings reinforce the importance of considering mindfulness as a complementary component of occupational safety interventions while recognizing that its effects may vary according to the nature of the work environment, the duration and intensity of the intervention, and the organizational conditions in which it is implemented.

In contrast, the present study incorporated mindfulness within a structured safety framework, emphasizing both individual awareness and collective responsibility. This integrated approach appears to have mitigated potential risks associated with excessive autonomy, as no increase in noncompliance was observed. Instead, mindfulness supported adherence to safety practices while maintaining individual agency.

The findings of this study have both theoretical and practical implications for occupational safety management in high-risk industrial settings. From a theoretical perspective, the study contributes to the growing body of research on mindfulness in occupational safety by demonstrating that mindfulness can complement Behavior-Based Safety (BBS) through the incorporation of cognitive and emotional processes into a predominantly behavioral safety framework.

The significant improvement in attitudes toward occupational risk and accident prevention suggests that addressing workers' attention, awareness, and emotional regulation may strengthen the psychological foundations underlying safe behavior. From a practical perspective, the findings support the feasibility of incorporating a mindfulness-based intervention, enhanced by virtual reality (VR), into existing BBS programs rather than replacing established behavioral safety practices. This combined approach may provide organizations with an additional tool for strengthening safety attitudes and promoting greater awareness of occupational risks, particularly in high-risk work environments.

Furthermore, the use of VR offers an experiential component that can facilitate realistic and immersive safety training while avoiding exposure to actual hazardous situations. Although the reduction in work-related stress did not reach statistical significance, the observed direction of change suggests that mindfulness-based safety interventions may have potential beyond safety attitudes, warranting further investigation through larger samples and longer intervention periods. Overall, the main contribution of this study lies in proposing and empirically evaluating an integrated safety framework that combines behavioral (BBS), cognitive-emotional (mindfulness), and experiential (VR) dimensions, thereby expanding conventional approaches to occupational accident prevention.

Despite the findings, this study has limitations that should be considered. First, the 30% dropout rate in the experimental group (attributed mainly to minor external) may have affected the statistical power to detect more subtle differ-fences. Furthermore, due to the active nature of the interventions, a double-blind design was not feasible. Although the risk of bias was mitigated by masking the hypothesis for participants and analyzing data based on protocol adherence, individual expectations may have influenced the results. It is suggested that future studies use active placebo control groups (e.g., neutral VR content) to better control for confounding variables.

Why did mindfulness not significantly reduce work-related stress? Although a reduction in work-related stress was observed following the intervention, this difference did not reach statistical significance. This finding does not necessarily contradict previous evidence regarding the benefits of mindfulness in the workplace; rather, it may be related to the characteristics of the present intervention and the multifactorial nature of occupational stress. First, the relatively small sample size (*n* = 34) may have limited the statistical power to detect small or moderate effects. Second, the intervention was relatively; therefore, it is possible that nine sessions were sufficient to modify attitudes toward risk prevention but not to produce statistically detectable changes in a more complex and potentially more stable construct such as work-related stress. Although systematic reviews have found favorable effects of mindfulness-based interventions on employee stress, they have also reported considerable heterogeneity across interventions, populations, and outcomes assessed (43).

Likewise, work-related stress does not depend exclusively on individual psychological resources but is also influenced by job demands, available resources, and organizational characteristics; therefore, an intervention primarily focused on the individual may not be sufficient to substantially modify stressors arising from working conditions (44, 45). In this regard, the non-significant result may be interpreted as a relevant finding suggesting that, to achieve more consistent reductions in work-related stress, mindfulness-based strategies should be complemented by interventions targeting organizational and psychosocial factors in the workplace. This interpretation is consistent with the perspective that mindfulness constitutes a potentially useful psychological resource, but not an isolated factor capable of explaining or independently modifying all sources of occupational stress.

## 5 Conclusions

The findings of this study demonstrate that the implementation of a mindful-ness-based program can have a significant positive impact on employees' attitudes toward occupational risk prevention and on several dimensions of work-related stress and job satisfaction. The results confirm that mindfulness training fosters safer work-place behaviors, greater personal responsibility, and reduced reliance on fatalistic or superstitious explanations of workplace accidents. These outcomes align with previous research suggesting that mindfulness enhances employees' awareness and emotional regulation, in turn promoting safer practices and improving overall wellbeing.

At the organizational level, mindfulness contributed to improvements in factors such as organizational climate, territory, leadership, and group support. By increasing self-awareness and empathy, the program fostered more collaborative and emotionally supportive work environments. Although mindfulness did not completely eliminate work-related stress, it played an important role in helping employees regulate stress levels and maintain emotional balance in demanding work contexts. This indicates that mindfulness functions as a management and adaptation mechanism rather than a full solution to chronic or structural stressors.

Comparison with the control group subjected to the Behavior-Based Safety (BBS) program revealed that the participants in that group experienced increased stress levels, possibly due to the cognitive and emotional demands of adapting to new behavior-al expectations. This contrast highlights the relative advantage of mindfulness-based approaches, which emphasize internal regulation and self-awareness rather than behavioral enforcement alone.

Importantly, this study also shows that integrating mindfulness practices within a structured safety climate framework can prevent potential drawbacks observed in other research—such as excessive autonomy or selective compliance with safety rules. The combination of mindfulness and collective safety reflection encouraged adherence to preventive measures without diminishing individual agency.

In conclusion, mindfulness-based interventions are a valuable tool for improving occupational safety attitudes and managing work-related stress. However, to maxim-size their long-term effectiveness, these programs should be accompanied by organizational strategies that address structural sources of stress and reinforce a shared culture of safety and wellbeing. Future research should continue exploring how mindfulness can be integrated with broader organizational development initiatives to strengthen both individual and systemic resilience in the workplace.

## Data Availability

The original contributions presented in the study are included in the article/supplementary material, further inquiries can be directed to the corresponding author.
